# Differences in fruit and vegetable intake and determinants of intakes between children of Dutch origin and non-Western ethnic minority children in the Netherlands – a cross sectional study

**DOI:** 10.1186/1479-5868-3-31

**Published:** 2006-09-22

**Authors:** Saskia J te Velde, Marianne Wind, Frank J van Lenthe, Knut-Inge Klepp, Johannes Brug

**Affiliations:** 1 Department of Public Health, Erasmus MC, University Medical Center Rotterdam, The Netherlands; 2 Department of Nutrition, Faculty of Medicine, University of Oslo, Norway

## Abstract

**Background:**

Fruit and vegetable consumption is low in the Netherlands and a key target in healthy diet promotion. However, hardly any information is available on differences in fruit and vegetable consumption between Dutch children and ethnic minority children. Therefore, the aim of present study was to determine differences in usual fruit and vegetable intake between native Dutch and non-Western ethnic minority children and to study differences in and mediating effects of potential psychosocial and environmental determinants.

**Methods:**

Ethnicity, usual fruit and vegetable consumption, psychosocial and environmental determinants and mothers' educational level were measured with a self-administered questionnaire during school hours in primary schools in Rotterdam, the Netherlands. Complete data was available for 521 10–11 year-old-children, of which 50.5% of non-Western origin. Differences between the groups regarding potential determinants and fruit and vegetable intake were assessed with Mann Whitney tests or multiple regression analyses. Multiple regression analyses were also conducted to assess mediating effects.

**Results:**

Ethnic minority girls ate fruit more frequently (1.41 ± 1.0 times/day) than Dutch girls (1.03 ± 0.82 times/day); no differences in frequency of intake were found for vegetables or among boys. Ethnic differences were found for almost all potential determinants. The Dutch children reported lower scores on these determinants than the ethnic minority children, except for perceived self-efficacy and barriers to eat fruit and vegetables. Knowledge of recommendations and facilitating behaviors of the parents mediated the association between ethnicity and fruit consumption among girls.

**Conclusion:**

Ethnic minority girls in the Netherlands appear to have more favorable fruit intakes than Dutch girls, and ethnic minority children in general show more positive prerequisites for fruit and vegetable consumption. Interventions addressing multi-ethnic populations of children must take such differences into account.

## Background

About 10% of the population in The Netherlands is of recent non-Western immigrant origin, with Turkish people forming the largest ethnic minority group [1]. In the major cities, this percentage is more than 30% [1]. The prevalence of chronic diseases and mortality varies between the ethnic minority groups and the native population [2-6]. Ethnicity may reflect biological, cultural, linguistic, religious, personal history as well as behavioral differences [7-11]. Common explanations for the increased risk for chronic diseases in non-Western immigrants is the fact that they more often have lower socio-economic position, accompanied by living in deprived areas, poorer working conditions, and more often engage in health risk behaviors such as smoking, sedentary behaviors, and high fat intakes [3,12-14]. However, there is some evidence that some of the larger immigrant groups in the Netherlands have healthier diets. Their diets may be related to Mediterranean diets with, for example, higher consumption of fruit and vegetables. High fruit and vegetable intakes have been associated with lower risks for cardiovascular diseases, some forms of cancers [13,14] and obesity [15]. It has been shown that such specific ethnic dietary behaviors may be persistent in a new home country [16-18]. Nevertheless, others report decreasing intakes of fruit and vegetable in immigrants, related to the degree of acculturation [19-21].

Fruit and vegetable promotion is one of the key targets in healthy diet promotion in the Netherlands as well as world-wide [19-21]. There is strong evidence that fruit and vegetable consumption is too low in the Netherlands, and that it is declining especially in children and adolescents [22]. However, there is a dearth of information

on differences in fruit and vegetable intake between children from native Dutch and immigrant origin. Better insight in such differences may help to tailor health promotion interventions. For such ethnic tailoring of nutrition education interventions, insight in potential determinants or mediators of intake is necessary [23,24]. To explain health related behavior, social cognition theories are often used [25]. The model posits that behavior, such as fruit and vegetable consumption, is mainly influenced by cognitions such as attitudes, perceived behavior control, self-efficacy, health beliefs, or risk perceptions. Especially for children, who may have less autonomy in making dietary choices.

**Figure 1:**
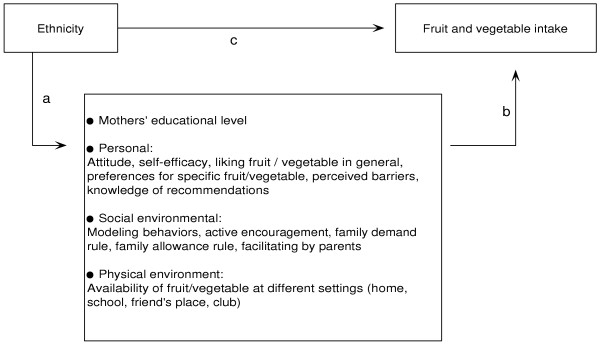
Conceptual model of the relationship between ethnicity and fruit and vegetable consumption and the potential mediators in this relationship.

The aim of the present study was to explore possible differences in fruit and vegetable intake between 10–11 year old children of Dutch origin on the one hand and children from non-Western immigrant origin on the other. Furthermore, differences between these groups of children in potential personal and environmental determinants of fruit and vegetable intake were studied. Moreover, we investigated if differences in fruit and vegetable intake, if any, were mediated by these potential personal and environmental determinants of fruit and vegetable intake or the mother's educational level (see Figure 1).

## Methods

### Design and subjects

The study described in this paper is based on the baseline data of the Dutch intervention study of the Pro Children Project, a cross- European study on fruit and vegetable intake among children [26]. As mentioned before, in the major cities, including Rotterdam, the percentage of people of non-Western origin is above 30% [1]. However, this percentage covers all age groups. The percentage of non-Western immigrants is much higher among the younger age groups, especially among primary school children. A recent report describes that at least 45% of the 9–10-year-old children are from non-Western origin [27]. Therefore, these Dutch data are well suited to explore some ethnic differences in fruit and vegetable intake and its determinants.

For the Dutch part of the Pro Children intervention study, data was collected among children from 24 schools, randomly selected in Rotterdam, the Netherlands. For the baseline measurements, the 10–11 year-old schoolchildren received a questionnaire, which they completed during school hours in the presence of a project worker. One school lesson (maximum 60 minutes) was used to complete the questionnaire that contained questions on demographics, fruit and vegetable intake, and personal and environmental determinants of fruit and vegetable consumption. Children received another questionnaire to take home for completion by one of their parents. Parents had to provide written informed consent for themselves and their child.

Initially, 76 schools were sampled of which 24 schools with 735 eligible students participated in the study. Because of illness or absence at the data collection school hour (n = 58), or because of lack of informed consent (n = 87), 599 children were included in the baseline measurements. Due to missing values (n = 22) and exclusion of the Western immigrant children (including immigrants from Indonesia and Japan) (n = 46), a maximum of 531 children was included in the analyses for the present study. Since some children had missing data for some of the variables, the number differed slightly between different analyses, as indicated in the relevant tables.

### Measures

Within the Pro-children project valid and reliable instruments were developed based on the theoretical framework, as described elsewhere [26,28,29]. Briefly, the theoretical framework was mainly inspired by Flay's Theory of Triadic Influences [30] and extended with the 'attitude, social influences, self-efficacy model' [31] and Bandura's Social Cognitive theory [32]. The framework proposes that the more distal determinants of fruit and vegetable intake can be found in the cultural, physical and social environment and that they in turn influence the more proximal personal factors such as attitude, social-influences and self-efficacy. The framework also recognizes the role of the environment, such as availability. French et al proposed that physical environmental factors, such as the availability and accessibility of foods, and social environmental factors may be important additional determinants of eating behaviors [33].

Both the intake part of the questionnaire as well as the determinants part of the questionnaire were pilot tested and validity and reliability were assessed [28,29].

The children themselves answered questionnaires on demographic variables, children's fruit and vegetable intakes and personal, social and environmental determinants of fruit and vegetable intake. The parents completed questionnaires on country of origin and educational level.

### Country of origin/ethnic background

In line with Dutch government definitions, children were considered being of Dutch origin when both parents were born in the Netherlands. From now on, we will use the term 'Dutch' to refer to these children. Children who had at least one parent born outside Europe (including former Soviet Union and Yugoslavia), North-America, Oceania, Indonesia and Japan were considered as non-Western immigrant children [1]. We will use the term 'non-Western immigrant children' to refer to these children, even though most of them have the Dutch nationality. Data from children from Western immigrants (at least one parent born outside the Netherlands but in one of the aforementioned 'Western' countries; N = 46) were not considered in the present study.

### Fruit and vegetable intake

Fruit and vegetable intake were assessed by food frequency questions (FFQ) and by the number of portions consumed on the day prior to the day of investigation (24-hour recall). Frequency of fruit intake was assessed with the frequency question "How often do you usually eat fresh fruit. Frequency of vegetable intake was assessed with separate questions for salad, raw or grated vegetables, and boiled or otherwise heated vegetables (subjects were only taken into account when at least two of these questions were completed). All four questions had eight response alternatives ranging from 'never' to 'more than twice a day'. With this data total fruit and vegetable intake were calculated as times per day.

Both the FFQ and the 24-hours recall were pilot-tested and validity and reproducibility were assessed in the Pro Children consortium countries. Correlations between the frequency questionnaire answers regarding total fruit and total vegetable intake and the reference method (the 7-day food record) varied between 0.38 and 0.53. Correlations for total fruit and total vegetable intake between the test and the re-test measurement in six countries varied between 0.47 and 0.76 [29]. These results were better than what is published so far on validity and reproducibility of food frequency questionnaires for children [34]. Based on this formal testing of the child instruments in Belgium, Denmark, Iceland, Norway and Portugal and comparisons with other studies, it was concluded that the questionnaire instrument was valid and reliable in giving national group means of vegetable and fruit intake among 11-year old children as well as ranking children by intake [29].

### Potential determinants of fruit and vegetable intake

An elaborate description of the development, reliability and validity of the questionnaire and the exact formulation of the questions has been published previously [28,35]. English and national versions of the questionnaire can be found at the Pro Children website [36]. The constructs and response categories are provided in the Appendix of this paper. Potential correlates of fruit and vegetable intake within the domain of personal factors included attitude towards eating of fruit and vegetables, general self-efficacy to eat fruit and vegetables, liking fruit and vegetables in general, preferences for specific fruit and vegetables, and perceived barriers to prevent eating fruit and vegetables, knowledge about recommended intake levels. Social environmental factors included modeling behavior of friends and parents, active parental encouragement, whether parents facilitate intake of fruit and vegetables by cutting them for their child (parental facilitation), and whether parents demand that their child eat fruit and vegetables (parental demand) or allow their child to eat as much fruit and vegetables as they want to (parental allowance). Physical environmental factors included perceived availability of fruit and vegetables at home, school, at friends' homes, and at the (sports)club. For all these questions responses ranged from 'fully disagree' (-2) to 'fully agree' (2) with higher values reflecting more positive determinants for high fruit or vegetable intake. The response options for knowledge were recoded into a dichotomous variable: less than the recommended intake levels versus the recommended intake levels or more. For constructs measured with more than 1 item, scale scores were computed as the mean of the relevant items after sufficient internal consistency was established. To compute scales including more than 1 item, at least 50% of the questions had to be answered and only children that completed at least 67% of the determinant part of the questionnaire were included for analyses.

The questionnaire aimed at measuring a broad range of potential determinants for fruit and vegetable intake separately. Consequently some constructs were measured by a single item in order to keep the questionnaire as short as possible. This was necessary because the children had only one school hour to complete the questionnaire and are also not capable of concentrating on the questionnaire much longer. Despite the use of single item constructs, this questionnaire was found to be reliable and valid for assessing personal, social and environmental factors of potential influence on fruit and vegetable intake in 10–11 year olds [28]. Most constructs showed good to very good intra-class correlation coefficients (ICC) (>0.60) for test-retesting, the remaining constructs (6 out of 30) showed acceptable ICC's, ranging between 0.50–0.59. Cronbach's alpha's were mostly moderate to high (range 0.52–0.89), except for the general self-efficacy scale (for fruit α = 0.42, for vegetable α = 0.49) [28].

Because the self-efficacy constructs showed low Cronbach's alpha's, we used the two items separately in the analyses (difficulty item and ability item).

### Mother's educational level

As an aspect of socio-economic status, educational level of the mother was used which was assessed by a questionnaire completed by the parents. Answer possibilities for educational level were based on the number of years of education: less than 7 years, between 7 and 9 years, between 10 and 12 years and more than 12 years. Data on the mothers' educational level was available for 424 of the 531 children (79.8%). More Dutch mothers reported their educational level (89.9%) than non-Western immigrant mothers (60.8%, p < 0.001), but the children of non-responders on educational level did not differ from the responders regarding sex, fruit- and vegetable intake.

### Data analyses

Descriptive statistics were calculated for the independent variables, sex, age, and mother's educational level, and differences between ethnic groups in these variables were explored with Students' t-test or Chi^2^-test.

Continuous dependent variables (frequency of fruit and vegetable intake and their potential determinants) were checked for normality. Frequency of fruit intake was considered as normally distributed, while frequency of vegetable intake was adjusted for positive skewness with a log transformation ln(x+1). Number of portions of fruit and vegetable consumed at the day prior to the assessment were not normally distributed. Log transformations did not solve this. To assess associations between ethnicity and frequency of fruit and vegetable intake, multiple linear regression analyses were performed with ethnicity as an independent dichotomous variable (native Dutch = 0, non-Western immigrant = 1) and either fruit or vegetable intake as dependent variables. In all analyses the Dutch children were coded as the reference category (0). Therefore, the regression coefficient in the linear regression analyses represents the difference between the non-Western immigrants and the Dutch when fruit consumption is addressed. For frequency of vegetable consumption, the presented regression coefficient is the back transformed regression coefficient from the analyses with the transformed vegetable intake, and therefore reflects the ratio between the groups (ln(y_1_) - ln(y_2_) = ln(y_1_/y_2_)). Analyses were adjusted for sex. Socio-economic factors associated with fruit and vegetable intake could also be associated with ethnicity and hence confound apparent ethnic differences. Therefore, in a second model educational level was included to assess possible confounding. For the regression analyses mother's educational level was dichotomized, as follows: educational level up to 9 years (0) versus 10 or more years (1) of education. The variable was dichotomized in order to reduce the number of parameters to be estimated in the regression models and because some categories included only a small number of subjects.

The results from the FFQ were used in all analyses. Although results from the FFQ are well suited to rank children according their intake levels and to use in analytical analyses, they were not validated to measure actual intake levels [29]. Therefore, results from the 24-hour recall were used to verify potential differences in intake levels between the native Dutch children and the non-Western immigrant children.

Because these data were not normally distributed, differences were tested by the non-Parametric Mann-Whitney U test.

Most of the potential determinants for fruit and vegetable intake had a skewed distribution. Therefore, non-parametric Mann Whitney tests were conducted to study differences in potential determinants of fruit and vegetable intake between the groups.

Regression analyses were further used to identify mediation by the potential determinants. Baron & Kenny and MacKinnon describe criteria that must be met for a variable to be considered a mediator [37-39]. In the present study this implies that, (I) ethnicity must be independently associated with the potential mediator (path a, Figure 1); (II) ethnicity must be associated with intake (path c, Figure 1); (III) the potential mediator must be independently associated with intake (path b, Figure 1), and (IV) the association between ethnicity and intake must decrease substantially when adjustment is made for the potential mediator. To determine the associations between the potential mediators and intake, single, per domain and multiple mediator models, both controlled for ethnicity, were applied. The former refers to separate regression analyses for each potential determinant on the outcome, the per domain analyses includes all determinants from the same domain (for instance personal domain) while the latter refers to one regression model that includes all potential determinants at the same time.

When the association between ethnicity and intake becomes non significant after adjustment for the mediator, full mediation can be concluded; when the association decreases not fully, partial mediation can be concluded, while when a strengthening of the association between ethnicity and intake is observed, it indicates inconsistent mediation or a suppression effect [38].

For associations, the significance level was set at p < 0.05.

Data was analyzed using SPSS version 11.

## Results

### Characteristics

Slightly more than half of the children were from non-Western immigrants (N = 273, 51.4%, with relatively large groups with parents born in Turkey (N = 66, 12.4%), Morocco (55; 10.4%) and the Dutch former colonies Surinam and Netherlands Antilles (N = 51; 9.6%)). No differences between ethnic groups in distribution of gender or age were found. However, the mothers of the Dutch children had significantly more years of education (Table 1).

**Table 1:** Characteristics of the study population by ethnicity

	**Total population**		**Dutch**		**non-western immigrants**	
	N = 531		N = 258	48.6%	N = 273	51.4%
	
	mean		mean		mean	SD
age (years)	10.7	0.5	10.7	0.5	10.8	0.5
gender (# girls)	280	52.7%	133	51.6	144	54.8
Mother's educational level	N = 424	%	N = 232	%	N = 192	

less than 7 years	52	12.3	5	2.2	47	24.5
7–9 years	45	10.6	14	6.0	31	16.1
10–12 years	98	23.1	64	27.6	34	17.7
more than 12 years	229	54.0	149	64.2	80	41.7

### Fruit intake

On average, the children reported eating fruit 1.15 ± 0.9 times a day, which was significantly higher among girls (1.24 ± 0.96 times/day) than among boys (1.24 ± 0.96 versus 1.06 ± 0.91 times/day, p = 0.033). Forty-six percent of the children reported no daily fruit intake. Results from the 24-hour-recall showed also higher intakes for girls than for boys, but the difference was not significant (median = 1.5 (inter-quartile range (IQR) 0.5–3.0) portions/day vs 1.0 (IQR 0–3.0) portions/day).

The difference in fruit intake between the ethnic groups was different for boys and girls (sex * ethnicity interaction: b = 0.36; p = 0.028). Stratified analyses showed that the difference in fruit intake was only significant for girls with the immigrant girls having the highest intakes (1.41 ± 1.0 times/day) and the Dutch girls the lowest (1.03 ± 0.82 times/day). This difference decreased to 0.25 times/day (95% CI [0.002–0.50]) and became borderline significant (p = 0.050) after further adjustment for mother's educational level (Table 2). Also results from the 24-hour-recall showed higher intakes among the non-Western immigrant girls compared with the Dutch girls, although not statistically significant (median = 2.0 (IQR 0.5–3.0) portions/day vs 1.5 (IQR 0.5–3.0) portions/day).

**Table 2:** Results of multiple linear regression analyses for differences in fruit and vegetable intake between Dutch children and non-Western immigrant children

	Fruit intake				Vegetable intake		
		
		N	β^a^	95% CI	N	β^b^	95% CI
Model 1	boys	249	0.02	-0.21; 0.25			
	girls	280	**0.37**	0.15; 0.59	529	0.97	0.89; 1.04
Model 2	boys	196	-0.05	-0.34; 0.25			
	girls	227	**0.25**	**0.00; 0.50**	423	1.01	0.92; 1.11

Model 1 – adjusted for sex; Model 2 – Model1 + adjusted for mother's educational level (dichotomous)

^a^β reflects difference non-Western immigrants (1) versus Dutch (0)

^b^β reflects ratio Dutch: non-Western immigrants

### Vegetable intake

The median intake for the whole group was 1.28 times/day (inter-quartile range 0.57 – 2.0 times/day). No differences in (back transformed) mean vegetable intake between the groups were observed as presented in Table 2. Overall, 42.7% of the children reported that they did not eat vegetables everyday. No significant difference in this proportion was found between ethnic groups. Regarding the 24-hour-recall no differences were found between the Dutch and non-Western immigrant children (median = 1.0 (IQR 0.0–2.0) portions/day vs 1.0 (0.0–2.5) portions/day, respectively).

### Determinants of fruit and vegetable intake

Non-Western immigrants had higher scores than the native Dutch children on most potential determinants for fruit intake (Table 3). They reported more positive attitudes towards eating fruit, had higher scores on liking, reported more preferences, perceived more active encouragement and facilitation from their parents, and more availability at sports- or leisure times club. However, the Dutch children scored significantly higher on the difficulty item of the self-efficacy construct and perceived fewer barriers to eat fruit. A similar pattern was found for vegetable intake, as shown in Table 4.

**Table 3:** Mean scores on determinants for fruit intake

	Total			Dutch		non-Western		
	
	N	Mean	SD	Mean	SD	Mean	SD	p^a^
Attitude	528	1.36	0.77	1.26	0.81	1.46	0.73	**0.001**
Self efficacy								
Difficulty item ^1^	525	0.45	1.52	0.74	1.47	0.18	1.53	**p < 0.001**
Ability item ^1^	526	1.48	0.88	1.42	0.94	1.55	0.81	0.164
Liking	528	1.5	0.67	1.43	0.75	1.57	0.58	**0.026**
Preferences	527	1.24	0.65	1.16	0.71	1.32	0.58	**0.016**
Perceived barriers	526	-1.3	0.84	-1.41	0.77	-1.20	0.89	**0.005**
Correct Knowledge (%)	524	61		58		64		**0.173**

Modeling	528	0.96	0.82	0.91	0.83	1.01	0.81	**0.133**
Active encouragement	527	0.62	1.31	0.38	1.36	0.84	1.23	**0.000**
Demanding	516	0.56	1.18	0.54	1.2	0.59	1.16	**0.696**
Allowing	515	1.51	0.89	1.52	0.83	1.51	0.94	**0.531**
Facilitating	522	0.55	1.15	0.34	1.17	0.75	1.09	**0.000**

Home availability	527	1.2	0.65	1.24	0.6	1.15	0.69	**0.202**
School availability	518	-1.69	0.81	-1.77	0.69	-1.61	0.9	**0.031**
Availability at friends	503	0.51	1.28	0.61	1.22	0.42	1.33	**0.114**
Availability at club	406	-0.43	1.51	-0.59	1.46	-0.24	1.54	**0.019**

^1 ^answers were recoded in a way that higher scores reflect higher self-efficacy

^a ^p-value based on Mann-Whitney test between Dutch and non-Western immigrants

**Table 4:** Mean scores for determinants for vegetable intake

	Total			Dutch		non-Western		
	
	N	Mean	SD	Mean	SD	Mean	SD	p^a^
Attitude	529	1.16	0.87	1.03	0.89	1.27	0.83	**p < 0.001**
Self efficacy								
Difficulty item ^1^	527	0.25	1.55	0.51	1.48	0.00	1.57	**p < 0.001**
Ability item ^1^	526	1.08	1.18	1.04	1.19	1.13	1.16	0.328
Liking	528	0.87	1.03	0.76	1.07	0.99	0.98	**0.006**
Preferences	529	0.69	0.84	0.57	0.83	0.81	0.83	**0.001**
Perceived barriers	528	-1.15	0.96	-1.33	0.85	-0.97	1.02	**p < 0.001**
Correct Knowledge (%)	524	37		0.35	0.48	0.38	0.49	0.384

Modeling	529	0.97	0.84	0.87	0.90	1.06	0.77	**0.019**
Active encouragement	525	0.58	1.34	0.41	1.34	0.74	1.33	**0.001**
Demanding	530	0.59	1.16	0.57	1.14	0.62	1.19	0.607
Allowing	529	1.24	1.04	1.23	1.00	1.25	1.09	0.393
Facilitating	514	-0.08	1.34	-0.49	1.25	0.32	1.29	**p < 0.001**

Home availability	527	0.96	0.78	0.95	0.70	0.96	0.85	0.427
School availability	519	-1.65	0.87	-1.75	0.78	-1.56	0.95	**0.005**
Availability at friends	518	-0.09	1.45	-0.12	1.39	-0.06	1.52	0.626
Availability at club	413	-0.82	1.42	-1.00	1.33	-0.61	1.49	**0.008**

^1 ^answers were recoded in a way that higher scores reflect higher self-efficacy

^a ^p-value based on Mann-Whitney test between Dutch and non-Western immigrants

### Mediation analyses

Since only the association between ethnicity and fruit consumption was significant in girls, we explored mediation for this association. In regression models with single potential mediators, 12 variables were significantly associated with fruit intake: attitude, self-efficacy, knowledge, liking, preferences, perceived barriers, demanding rule, allowing rule, facilitation by parents, modeling, home availability and availability at friends. However, as a result of inter-correlation between these potential determinants (data not shown), in the per domain multiple mediator models only the difficulty item of the self-efficacy construct, knowledge, liking, demanding family rules, facilitation by parents, modeling, and home availability were significantly related to fruit consumption in girls. Mother's educational level was not significantly independently associated with fruit intake in girls.

In girls, the non-Western immigrant children had significantly higher scores on attitude towards eating fruit, knowledge of recommended intake levels, active encouragement by their parents and facilitation by their parents. The Dutch girls had significantly higher scores on the difficulty item of the self-efficacy construct for eating fruit. According to the criteria for mediators, the difficulty item of the self-efficacy construct, knowledge, and facilitation should thus be further explored as possible mediators of the association between ethnicity and fruit intake in girls. Table 5 shows the change in the regression coefficient for ethnic group when adjustments are made for these potential mediators. As can be seen, adding the difficulty item for self-efficacy to the model, the association between ethnic group and intake becomes stronger, which indicates so-called inconsistent mediation or suppression. So, actually the association between ethnic group and fruit intake is stronger than presented in Table 2, when not taking this difficulty item into account. When controlling for this self-efficacy item, thus assuming self-efficacy constant, the difference between the ethnic groups increases (0.511 times/day).

**Table 5:** Effect of adjustment for potential mediators in the association between ethnicity and fruit intake in girls.

Potential mediator	Original ^1^	After adjustment	%
'Difficulty' item of self-efficacy construct (n = 278)	0.381***	0.511***	34.1
Knowledge (n = 277)	0.398***	0.327**	-17.8
Facilitation (n = 275)	0.360**	0.281*	21.9
Knowledge & facilitation (n = 271)	0.381***	0.243*	36.2

^1 ^original regression coefficients differ because of different numbers in the analyses due to missing values on the potential mediators

*** p < 0.001 ** p = 0.01 * p = 0.05

When adding knowledge into the regression model a decrease in the regression coefficient from 0.398 to 0.327 (17.8% decrease) was observed. Adding facilitation by parents to the model, the regression coefficient decreased substantially (21.9%).

It was also determined whether mothers' educational level could be considered a mediator, since educational level was unequally distributed over the Dutch and immigrant groups (Table 1). However, as mentioned, it appeared not to be associated with fruit intake (β = -0.049 (95% CI -0.165 – 0.068)) and was therefore not eligible for further exploration as a possible mediator.

## Discussion

The aim of this study was to assess differences in fruit and vegetable consumption between Dutch children and children from non-Western immigrants living in Rotterdam, the Netherlands. Furthermore, we aimed to study differences in potential determinants of fruit and vegetable intake and whether these factors mediated the ethnicity-intake association.

In the present study, 51.4% of the participating children were of recent non-western immigrant origin. In 2001 the proportion of non-Western immigrant 6^th ^graders was at least 45% [27], thus current sample seems representative for the Rotterdam primary schoolchildren. Overall, the non-Western immigrant children had higher fruit intakes as assessed by frequency questions, caused by the higher intake of the girls in this group. Results from the 24-hour-recall also showed higher intake levels among non-Western immigrant children, although these differences were not significant. The relationship between ethnicity and fruit intake was significant in girls only. It is often reported that girls consume more fruits than boys [40,41], but we can only speculate why the association between ethnicity and intake differs between boys and girls.

Most studies reporting on differences in diet between immigrants and native populations have been conducted in the USA [16-18]. Only few investigations have focused on dietary intake of immigrants in the Netherlands or other European countries [8,42-44]. A previous Dutch study suggest that immigrants, or more specific, Turks and Moroccans, seem to eat more fruit than the native population [45]. However, the authors note that results from different studies are conflicting, based on non-published data from different local municipal health organizations. The main immigrant populations in The Netherlands originate from countries where fruit and vegetable consumption is usually higher than in the Netherlands. For example university students in Turkey reported a mean fruit intake of 1.83 ± 0.98 times/day for men and 1.79 ± 0.99 times/day for women [46], which is much higher than reported by the children, including the girls, in present study and also higher than Dutch adults report eating [47]. Food habits are considered important indicators of acculturation. According to a model proposed by Koctürk-Runefors staple foods are the last dietary component to change in a new country, while the accessory components will be the first to be replaced followed by complementary food items such as meat and vegetables [43,44]. So, accessory food from the new country, such as sweets and snacks, may be likely to displace traditional accessory food items such as fruit in a rather early stage. We observed no differences in vegetable intake between the ethnic groups and no differences in fruit intake between the ethnic groups among boys. This may be an indication that immigrant children are adopting Dutch dietary habits. Contradictory to this model, we found differences in fruit intake between the groups in girls only. Secondary analyses revealed that 85.4% of the children from recent immigrant origin were themselves born in the Netherlands and that 58.6% of them reported to speak Dutch at home. Those born in The Netherlands tend to have lower fruit intakes (1.21 times/day) than those born abroad (1.50 times/day; p = 0.111). Although this difference was not significant, which may be due to a lack of power because of the small groups, it suggests an ongoing process of adaptation to the Dutch food habits among immigrants. That immigrant girls have higher fruit intake, might be a result of less acculturation due to the way they are raised. Moroccan values and norms result into far more restrictions regarding the freedom of movement of females, compared to males. Dutch studies suggest an authoritarian child rearing style by Moroccan parents, especially towards their daughters [48], although this gender difference was not replicated in a recent study [49]. Turkish girls also have more tasks at home [50] and as a result of that they may have more similarities with the dietary behaviors of their parents than with their Dutch peers.

Knowledge of potential determinants of behavior is necessary for developing interventions, and information on determinants in ethnic groups is often lacking. The groups in our study differed on potential determinants at the personal and environmental levels, mostly in favor of the immigrant group. The Dutch children disagreed more with the statement 'it is difficult for me to eat fruit/vegetable every day' (item 1 of the self-efficacy construct) and reported to experience fewer barriers for eating fruit and vegetables. This was, however, not in accordance with their lower average consumption. In both groups separately, the difficulty item and perceived barriers were significantly correlated to fruit intake (data not shown), and no modification by ethnicity in associations between the difficulty item and perceived barriers with fruit intake was observed. Our results indicate that high self-efficacy may be a relevant factor, but not a sufficient factor for higher intakes. It can also be that the Dutch children think they already eat a lot of fruit and therefore experience no barriers or other difficulties preventing them to eat enough fruit. When asked to compare their own intakes to that of their peers, it appeared that indeed the Dutch children overestimated their fruit intake, while children from immigrant origin may underestimate their intakes compared to others. Optimism about own intake levels has been reported before for Dutch adolescents [45].

From the mediation analyses we observed a suppression effect by the self-efficacy item, which is in line with what is discussed before. It can be concluded that the association between ethnicity and fruit intake in girls is not mediated by self-efficacy, but that differences in intakes may be even larger in case of equal self-efficacy. Our results further provide evidence for mediation of the ethnicity-intake association by knowledge and facilitation by the parents, at least in girls. The fact that including these factors did not fully account for the difference in intake indicates that other, unknown mediators should be explored.

Adjustment for mother's educational level decreased the difference in fruit intake between Dutch and ethnic minority girls. However, mother's educational level was not independently associated with intake, and therefore not considered as a mediator. Mother's educational level was associated with ethnicity and therefore adjustment for that in the association between ethnicity and fruit intake can cause a decrease in the effect size.

A limitation of the present study is, of course, it's cross sectional design. We need longitudinal research to see whether changes in knowledge and facilitation are indeed associated with changes in the differences in intake between Dutch and non-Western girls.

Furthermore, our study population was derived from only one of the major cities in the Netherlands, and further research is needed to explore if our results are also valid for ethnic differences in other Dutch regions. Moreover there is need to verify whether our results hold for all ethnic minority groups. Because of the small numbers, we were not able to stratify analyses for different ethnic groups.

### Implications and conclusion

Although, we observed differences in almost all determinants between the two ethnic groups, we only found differences in fruit intake in girls. We identified knowledge and facilitation by the parents as potential mediators. This implies that ethnic differences in fruit intakes in girls can be diminished by targeting knowledge of recommended intake levels and parental facilitation among Dutch girls.

## Appendix

**Table:** Constructs and items of the determinant questionnaire

Constructs with items	Response categories
***Personal***	
Attitude To eat fruit/vegetables every day makes me feel good To eat fruit/vegetables every day gives me more energy	5 point scale from 2 = I fully agree to -2 = I fully disagree
Self efficacy It is difficult for me to eat fruit/vegetables every day If I decide to eat fruit/vegetables every day, I can do it	5 point scale from -2 = I fully agree to +2 = I fully disagree
Liking I like to eat fruit/vegetables every day Fruit/vegetables taste good	5 point scale from 2 = I fully agree to -2 = I fully disagree
Preferences fruit Which of the following fruits do you like or dislike, list of 12 fruits: apple, bananas, pears, oranges, tangerines, plums, peaches, melon, strawberries, grapes, cherries, kiwis	5 point scale from 2 = like very much to -2 = dislike very much and 0 = have not tried
Preferences vegetables Which of the following vegetables do you like or dislike? List of 12 vegetables: tomatoes, cucumber, salad, cabbage, spinach, leak green beans, onion, carrots, broccoli, cauliflower, green peas	5 point scale from 2 = like very much to -2 = dislike very much and 0 = have not tried
Perceived barriers fruit I do not eat fruit because it takes too much time to eat I do not eat fruit because I want to eat something else (e.g. sweets) I do not eat fruit because my fingers get greasy when I eat it I do not eat fruit because it gets squeezed in the schoolbag	5 point scale from 2 = I fully agree to -2 = I fully disagree
Perceived barriers vegetables I do not eat vegetables because it takes too much time to eat I do not eat vegetables because I am still hungry after having eaten them I do not eat vegetables because I want to eat something else (e.g. sweets) I do not eat vegetables because they get squeezed in the schoolbag	5 point scale from 2 = I fully agree to -2 = I fully disagree
Knowledge fruit How much fruit do you think you should eat to have a healthy diet?	1 = no fruit, 2 = 1–3 pieces per week, 3 = 4–6 pieces per week, 4 = 1 piece per day, 5 = 2 pieces per day, 6 = 3 pieces per day,7 = 4 pieces per day, 8 = 5 pieces per day or more recoded: correct knowledge = 5–8
Knowledge vegetables How many vegetables do you think you should eat to have a healthy diet	1 = no vegetables, 2 = 1–3 portions (serving spoons) per week, 3 = 4–6 portions per week, 4 = 1 portion every day,5 = 2 portions every day, 6 = 3 portions every day, 7 = 4 portions every day, 8 = 5 or more portions every day recoded: correct knowledge = 6–8
Perceived social environmental	
*Modelling* My mother eats fruit/vegetables every day My father eats fruit/vegetables every day My best friend eats fruit/vegetables every day	5 point scale from 2 = I fully agree to -2 = I fully disagree
Active encouragement My mother encourages me to eat fruit/vegetables every day My father encourages me to eat fruit/vegetables every day	5 point scale from 2 = I fully agree to -2 = I fully disagree
*Demanding* Do your parents demand that you eat fruit/vegetables every day?	5 point scale from 2 = yes, always to -2 = never
Allowing Are you allowed to eat as much fruit/as many vegetables as you like at home?	5 point scale from 2 = yes, always to -2 = never
Facilitating Does your mother or father usually cut up fruit/vegetables for you in between meals?	5 point scale from 2 = yes, always to -2 = never
Perceived physical environment	
Home availability If you tell at home what fruit/which vegetables you would like to eat, will it be bought? Are there usually different kinds of fruits/vegetables available in your home? Is/are there usually fruit/vegetables available at home that you like?	5 point scale from 2 = yes, always to -2 = never
School availability Can you get fruit/vegetables at school either by buying it or getting it for free?	5 point scale from 2 = yes, always to -2 = never
Availability at friends Can you get fruit/vegetables at you friends' house, when you spend the afternoon there?	5 point scale from 2 = yes, always to -2 = never
Availability at the club Can you get fruit/vegetables at the place where you have your leisure time activity (e.g. club, sports place) either by buying it or getting it for free?	5 point scale from 2 = yes, always to -2 = never

## Declaration of competing interests

The author(s) declare that they have no competing interests.

## Authors' contributions

SJtV performed the analyses and wrote the manuscript and incorporated input from all other authors on the manuscript. MW coordinated the data collection and implementation of the study and contributed in writing the manuscript. FJvL advised on the analyses and commented on drafts of the manuscript. KIK developed and directed the overall study and provide critical comments on the manuscript. JB developed and directed the overall study, and provided critical comments on the manuscript. All authors have read and approved the final version of the manuscript.
